# Healing Spinal Cord Injuries

**DOI:** 10.1371/journal.pbio.1000400

**Published:** 2010-06-22

**Authors:** Robin Meadows

**Affiliations:** Freelance Science Writer, Fairfield, California, United States of America

Unlike those in the periphery, nerve fibers in the central nervous system (brain and spinal cord) do not recover from traumatic injury. This makes disabilities from spinal cord damage permanent, with the severity depending on the location of the cord injury. Neck injuries can paralyze the torso and limbs, while lower back injuries can impair movement below the waist. The spinal cord is a bundle of long, thin fibers called axons that connect the brain to the body, and regrowth of these axons can be spurred by a variety of pharmacological treatments. In an effort to understand the basic mechanisms of axon regrowth, Ping Yip and colleagues report how a protein called neuronal calcium sensor-1 (NCS1) can help repair central nervous system (CNS) damage in rats.

Previous studies have linked NCS1 to neuron survival, as well as to the outgrowth or sprouting of neuronal processes. This sprouting can occur on axons, which transmit neuronal signals, and on dendrites, which receive signals. Yip and colleagues have previously shown that axons can regenerate in CNS neurons that overexpress retinoic acid receptor β2, a protein that regulates cell growth, and also observed that this regeneration is accompanied by a rise in NCS1 protein.

Here, Yip and colleagues further test the relevance of NCS1 to axonal regrowth in both cultured neurons and living rats. They begin by developing a method of increasing the expression of NCS1, and confirming this overexpression triggers sprouting in cultured neurons from adult rat brains. After treatment with a viral vector carrying NCS1 and green fluorescent protein (GFP), these neurons expressed five times the normal amount of NCS1 and sprouted abundantly. Labeling with a dendritic marker called microtubule associated protein 2 revealed sprouting on both axonal and dendritic projections. In contrast, untreated neurons hardly sprouted at all.

To gain insight into the mechanism of NCS1-induced sprouting, the researchers investigated the role of a pathway (P13K/Akt) that regulates cell growth and proliferation and increases neuron survival. They found that cultured neurons that overexpress also have high levels of phospho-Akt protein, indicating activation of the P13K/Akt pathway. Furthermore, blocking this pathway in NCS1-transduced neurons led to a drop in both phospho-Akt levels and neurite sprouting.

To see if these findings also hold for CNS neurons in living rats, the researchers injected the NCS1-GFP vector into the region of the cerebral cortex that controls limb movements for only one side of the body. The right half (or hemisphere) of the cortex contains neurons that control movement on the left side of the body, while the left hemisphere controls the right side of the body. Axons from each hemisphere of the cortex form the pyramidal tract in the base of the brain and then enter the spinal column. Like the brain, the spinal cord also has two halves, each of which controls one side of the body. Three weeks after the NCS1-GFP vector injection, the neurons and axons expressing high NCS1 levels were completely GFP-labeled all the way from the cortex to the spinal cord. The researchers then severed the pyramidal tract on the other side, denervating the untreated half of the spinal cord while leaving the treated half intact. Six weeks later, nerve fibers from the intact side of the spinal cord had extended into the injured side, confirming that NCS1 overexpression boosts axon sprouting in whole animals as well as in cell culture.

To determine whether this new sprouting actually translated into rescue of motor behavior, the researchers next asked how well NCS1-transduced rats could use their limbs on the injured side. One behavioral test required rats to use their forelimbs to reach for and grasp food pellets. Two days after their spinal cord injuries, the NCS1-transduced rats could hardly get food pellets with their affected forelimbs. But within 21 days, they grabbed food pellets as handily as uninjured rats. The other behavioral test assessed how well rats could walk on the wires of a mesh. Soon after their spinal cord injuries, NCS1-transduced rats were quite clumsy at navigating the grid with their affected limbs. But within 21 days, they were as surefooted as uninjured rats.

Finally, the reserachers tested whether NCS1 transduction after spinal injury would also lead to substantial axon regrowth and behavioral recovery. They found that NCS1 overexpression has much the same benefits when begun two days after injury as when begun beforehand. Encouragingly, these benefits include new fibers extending from the intact side of the spinal cord into the injured side as well as regeneration of fibers on the injured side. The combination of anatomical and behavioral recovery makes this work particularly promising, suggesting that therapies to increase NCS1 levels may someday help people recover from spinal cord injuries.

**Yip PK, Wong L-F, Sears TA, Yáñez-Muñoz RJ, McMahon SB (2010) Cortical Overexpression of Neuronal Calcium Sensor 1 Induces Functional Plasticity in Spinal Cord Following Unilateral Pyramidal Tract Injury in Rat. doi:10.1371/journal.pbio.1000399**

